# Outcomes That Matter in Gastrointestinal Cancer Care: A Focus Group Study of Patient‐Centred Outcomes Identified by Patients and Care Partners

**DOI:** 10.1111/hex.70835

**Published:** 2026-08-21

**Authors:** Anna Gombay, Anna Ding, Alyson Mahar, Amy T. Hsu, Lesley Gotlib Conn, Jessica Armah, Ekaterina Kosyachkova, Julie Deleemans, Claire Ludwig, Allia Karim, Natalie Coburn, Julie Hallet

**Affiliations:** ^1^ Evaluative Clinical Sciences Sunnybrook Research Institute Toronto Ontario Canada; ^2^ Health Quality Program, School of Nursing Queen's University Kingston Ontario Canada; ^3^ Ottawa Hospital Research Institute Ottawa Ontario Canada; ^4^ Bruyère Research Institute Ottawa Ontario Canada; ^5^ Institute of Health Policy, Management and Evaluation University of Toronto Toronto Ontario Canada; ^6^ Stomach Cancer Foundation of Canada Toronto Ontario Canada; ^7^ Cumming School of Medicine University of Calgary Calgary Alberta Canada; ^8^ School of Nursing, Faculty of Health Sciences University of Ottawa Ottawa Ontario Canada; ^9^ Reserca Toronto Ontario Canada; ^10^ Department of Surgery University of Toronto Toronto Ontario Canada; ^11^ Division of Surgical Oncology Odette Cancer Centre – Sunnybrook Health Sciences Centre Toronto Ontario Canada

**Keywords:** care partners, core outcome set, focus groups, gastrointestinal cancer, patient and public involvement, patient‐centred outcomes, risk communication

## Abstract

**Background and Objective:**

Gastrointestinal (GI) cancers pose a substantial burden on patients and care partners, yet outcomes research and clinical tools remain heavily weighted toward clinical endpoints over patient‐centred outcomes (PCOs). Without standardised PCOs, risk communication and shared decision‐making may revolve around endpoints misaligned with patient priorities. We aimed to identify PCOs meaningful to patients with GI cancer and their care partners.

**Design, Setting and Participants:**

We conducted a qualitative focus group study with adult patients with a GI cancer diagnosis and their care partners, recruited from a tertiary cancer centre and patient partner organisations in Canada. Focus groups used open‐ended questions and hypothetical patient personas to elicit PCOs. Two researchers independently extracted PCO concepts from verbatim transcripts and synthesised them using a deductive‐inductive clustering approach informed by thematic analysis and the patient‐centred care framework.

**Results:**

Overall, 15 patients and 13 care partners participated. We extracted 254 PCO concepts and consolidated them into 55 unique PCOs organised under 7 parent clusters: care experience (31% of mentions), psychosocial (25%), treatment (16%), lifestyle (12%), healthcare utilisation (8%), functional status (4%), and symptoms (3%). Six PCOs accounted for approximately half of all mentions: communication, care access, information access, treatment understanding, coping abilities, and finances. Patients most often raised psychosocial PCOs, whereas care partners more frequently mentioned functional status and symptom PCOs. Treatment‐related PCOs were a mutual concern.

**Discussion and Conclusions:**

Patients and care partners identified 55 PCOs spanning dimensions not consistently captured in existing GI cancer instruments. These PCOs can be integrated in the development of a core outcome set for patient‐centred risk assessment and individualised prediction in GI cancer care.

**Patient and Public Contribution:**

Three patient partners with lived experience of cancer contributed to the study as co‐investigators. They informed the focus group guide and its sequencing, advised on the language of recruitment materials, and refined data analysis and interpretation. Patients and care partners also took part as focus group participants.

## 1 Introduction

Gastrointestinal (GI) cancers are among the most common and deadly cancers worldwide [1, 2]. Beyond their high incidence and mortality, they place a profound burden on patients and care partners [3, 4]. Treatments are often invasive and prolonged, which can lead to substantial physical symptoms, emotional distress, and disruptions to daily life [5, 6, 7, 8]. Amidst these challenges, patients and care partners must frequently navigate complex treatment decisions, weighing potential benefits and risks and interpreting how these decisions impact important aspects of their lives.

Supporting patients and care partners through GI cancer requires a nuanced understanding of how they prioritise and value different care outcomes. While clinical outcomes quantify biological responses to care, patient‐centred outcomes (PCOs) can contextualise clinical results within the realities of living with cancer [9]. It is crucial to consider both types of outcomes to provide balanced care. However, contemporary research and clinical tools tend to be heavily weighted towards clinical outcomes, such as tumour response and survival, over PCOs such as personal independence or family functioning [10, 11, 12, 13]. Without standardised PCOs, evidence on patient centred care cannot be easily compared or synthesised across studies. This limits our ability to translate evidence into real‐world clinical practice and ensure patient‐valued outcomes meaningfully influence decision making in cancer care.

Outcome data form the foundation of risk assessment and communication in treatment decisions; they directly influence how patients, care partners, and clinicians evaluate options and make decisions about care. When outcomes data do not reflect what people value most, risk communication may revolve around endpoints that are misaligned with patient priorities and have repercussions on patient experience and care. Indeed, risk communication grounded in outcomes that matter to patients is associated with reduced decisional conflict and regret [14, 15]. A patient‐centred approach to outcome measurement is therefore essential to advancing both risk assessment and personalised risk communication within clinical practice in oncology.

Developing a core outcome set (COS)—an agreed minimum set of outcomes to be measured and reported for a given condition—is a common approach to standardising outcomes of importance for a specific disease population [16]. To ensure a COS reflects outcomes most meaningful to its intended audience, it must integrate evidence from both existing literature and from those with lived experiences of a specific disease [17]. To that end, the aim of this study was to identify PCOs that are meaningful to patients with GI cancer and their care partners, with a view to inform the development of a COS for patient‐centred risk assessment and prediction in GI cancer care.

## 2 Methods

### 2.1 Study Design

We used focus groups to elicit PCOs, defined as healthcare outcomes that align closely with patients' individual needs and preferences, directly from patients and care partners with lived experience of GI cancer [9]. This study was approved by the institutional Research Ethics Board (#5195; institution name withheld for peer review) and conforms to the Declaration of Helsinki; all participants provided written informed consent. We report the study following the Consolidated Criteria for Reporting Qualitative Research (COREQ) and the GRIPP2 short‐form checklist for reporting patient and public involvement (Supporting Information: Table S1).

### 2.2 Sampling and Recruitment

Individuals were eligible if they were adults (≥ 18 years old) with a GI cancer diagnosis or providing care for an individual with a GI cancer diagnosis and were able to participate in English‐language focus groups.

We recruited patients from outpatient GI cancer clinics at a single tertiary care cancer centre in Canada, and care partners through patient referral (i.e., patients referred their own care partners) and two patient partner organisations (names withheld for peer review). We enroled participants between March and December 2024, using purposive sampling to capture diversity across age, gender, cancer site and stage.

### 2.3 Data Collection

An experienced qualitative researcher conducted semi‐structured virtual focus groups in English, along with one member of the research staff who took notes. Focus groups were planned for approximately five participants each. Where scheduling constraints prevented convening larger groups, we proceeded with smaller groups (minimum two participants) to facilitate participation.

Two to five participants joined at a time. Focus groups ran for approximately 90 min each.

The focus group guide included open‐ended questions and structured prompts to balance key topics with participant‐led discussions. Questions were organised into three domains: (1) initial concerns immediately following diagnosis; (2) decision‐making around treatment; and (3) expectations for the future. To encourage discussion that was both personally reflective and broadly applicable, participants were asked to respond from the perspective of two hypothetical patient personas (Supporting Information: Figure S1). These personas were presented with visual profiles and accompanying narratives describing their symptoms, diagnoses, prognoses, and treatment options. Participants received the personas and focus group questions by email 1 day prior to their scheduled focus group to allow time for reflection.

Sessions were audio‐recorded, transcribed verbatim, and reviewed by the focus group moderator and research staff after each group to iteratively refine the facilitation guide. Focus groups continued until additional data collection no longer yielded new themes, insights, or meaningful conceptual information relevant to the research question. Repeat interviews or group discussions were not carried out and the transcripts were not returned to participants.

### 2.4 Data Analysis

Focus groups were transcribed using human transcription. Two researchers independently read each transcript and extracted PCO concepts mentioned by participants. A PCO concept was considered eligible if it met one of the following criteria: (1) the participant introduced the PCO concept independently, without being prompted by the facilitator; (2) the participant elaborated on a PCO concept initially prompted by the facilitator, providing detail or context beyond a simple affirmation (e.g. 'I agree', 'sure') of its relevance or importance.

**Figure 1:**
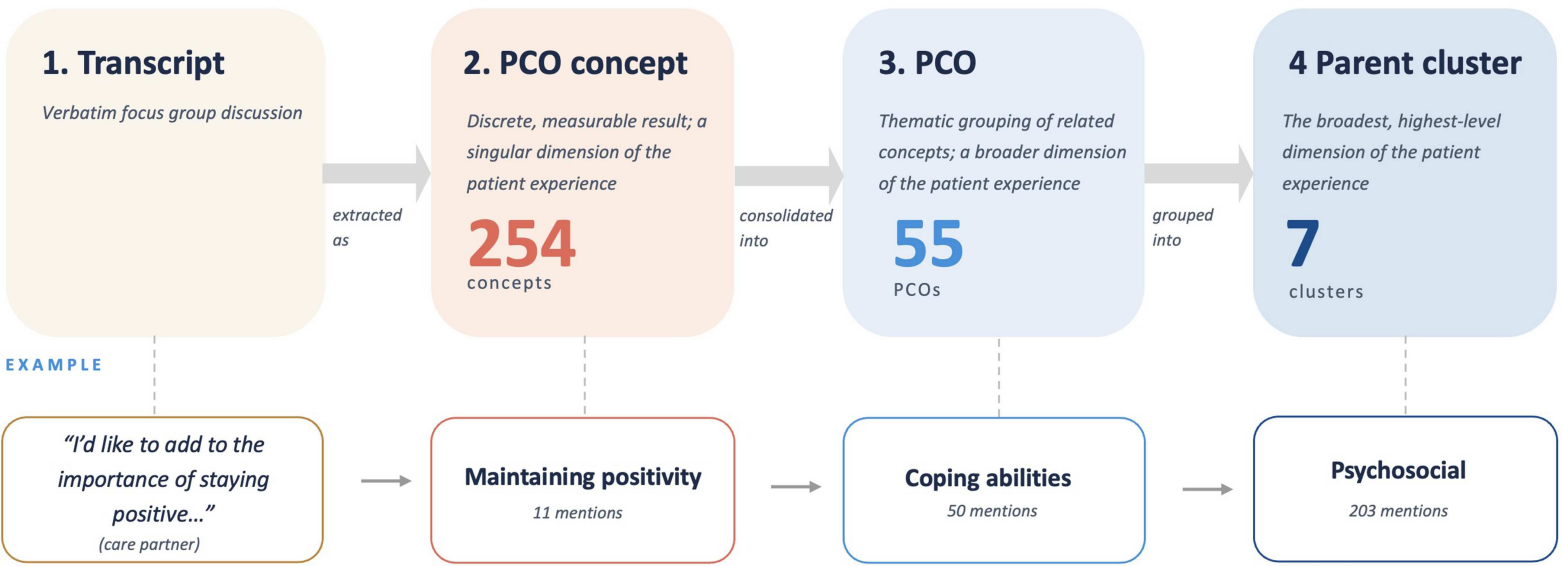
Data analysis schematics—extraction and classification process for patient‐centred outcomes (PCOs). The worked example traces one lineage from a care partner's statement to the cluster level.

We used a deductive‐inductive clustering approach informed by the principles of thematic analysis and the patient‐centred care framework [18, 19, 20, 21, 22]. We identified PCO concepts reflecting singular dimensions mentioned by participants (e.g., 'maintaining positivity', 'maintaining hope', 'managing stress'). We then synthesised PCO concepts into PCOs (e.g., 'coping abilities'). Finally, we nested each PCO under overarching parent clusters representing the highest‐level dimensions of the outcomes (e.g., 'psychosocial') grouping concepts into clusters based on thematic similarity until no further meaningful grouping was possible. Ultimately, we organised PCOs into a three‐level classification system (Figure 1). We counted the frequency of each PCO mention overall and broken‐down by type of participant (patient or care partner) [23, 24, 25]. This is reported as an absolute number (n) with percent (%) of total mentions.

### 2.5 Patient and Public Involvement

We worked with three patient partners with lived experience of cancer who were engaged as co‐investigators from the study inception with the aim of grounding the study goals and design, conduct, and interpretation in lived experience. They contributed to the development and sequencing of the focus group guide to ensure questions allowed sufficient time for domains most relevant to patients, advised on recruitment language to ensure terminology was clear, respectful, and sensitive, and refined how we defined, identified, and categorised PCOs throughout analysis. Patient partners were members of the research team and did not participate in focus groups or transcript coding. Their involvement influenced the study substantively, most notably the breadth of what we counted as a PCO.

## 3 Results

### 3.1 Participants

Between March and December 2024, we enroled 15 patients and 13 care partners, with focus groups of 2–5 participants each (Table 1). Focus groups continued until no new PCO concepts emerged across successive sessions. Primary cancers included gastric, colorectal, hepatic and pancreatic cancers.

**Table 1:** Participant characteristics.

		Patients (*n* = 15)	Care partners (*n* = 13)
**Demographic characteristics**			
Age (years old) ‐ average, range		58 (33–75)	62 (36–76)
Gender	Female	10 (67.7%)	9 (69.2%)
	Male	5 (33.3%)	4 (30.8%)
Race	White/Caucasian	9 (60%)	11 (84.6%)
	Black/African American	3 (20%)	2 (15.4%)
	Other	3 (20%)	—
Ethnicity	Canadian	6 (40%)	5 (38.5%)
	European	—	5 (38.5%)
	Multiple	4 (26.7%)	—
	Other	5 (33.3%)	3 (23.0%)
**Patient characteristics**			
Cancer type	Multiple sites	6 (40%)	—
	Pancreatic	4 (26.7%)	
	Other	5 (33.3%)	
Disease stage at diagnosis	II–III	3 (20%)	
	IV	6 (40%)	
	Unknown	6 (40%)	
Treatment	Chemotherapy	9 (60%)	
	Chemotherapy + radiation therapy	3 (20%)	
	Other	3 (20%)	
Surgery	Yes	8 (53.3%)	
	No	7 (46.7%)	
**Care partner characteristics**			
Relationship to patient	Spouse	—	10 (76.9%)
	Sibling		3 (23.1%)
Duration of care support prior to study	> 6 years		6 (46.2%)
	1–6 years		4 (30.7%)
	< 1 year		3 (23.1%)
Time commitment as a care partner	24/7		8 (61.5%)
	< 24/7		5 (38.5%)

*Note:* Values showing *n* (%) unless otherwise specified.

### 3.2 PCOs Identified

**Figure 2:**
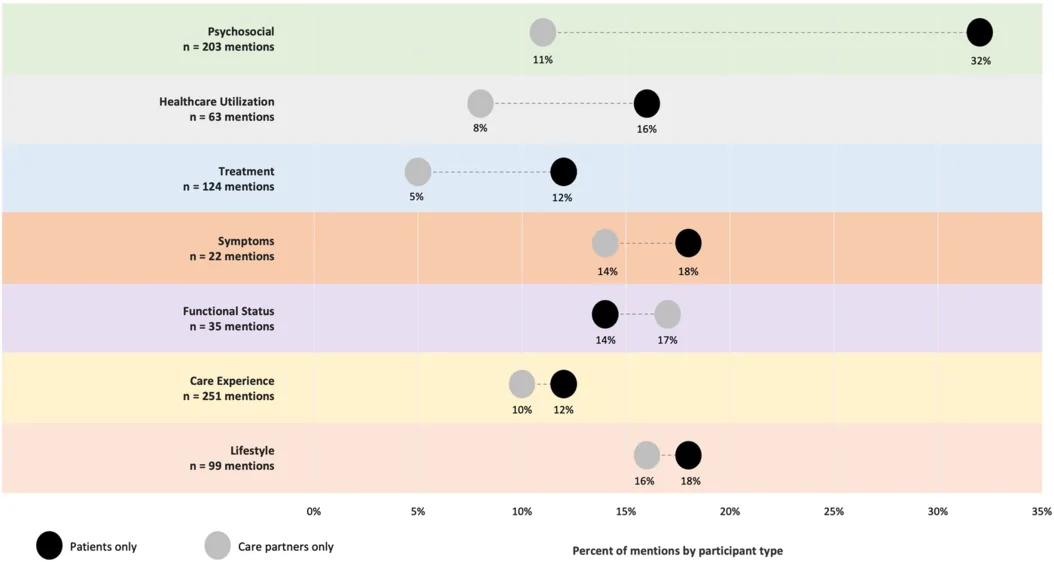
Patient‐centred outcomes (PCO) mentions by type of participant (patients and care partners).

We extracted 254 PCO concepts from the focus group transcripts and consolidated them into 55 unique PCOs organised under 7 parent clusters (Figure 1 and Table 2). The parent clusters were care experience (31% of mentions), psychosocial (25%), treatment (16%), lifestyle (12%), healthcare utilisation (8%), functional status (4%) and symptoms (3%) (Figure 2). Six PCOs together accounted for approximately half of all mentions: communication (11.7%), care access (9.5%), information access (7.7%), treatment understanding (6.4%), coping abilities (6.3%) and finances (5.0%). The full list of 55 PCOs with mention counts is presented in Table 2.

**Table 2:** Hierarchical classification of identified patient‐centred outcomes (PCOs) with underlying concepts.

Parent cluster	Patient‐centred outcome	Mentions (*n*)	% of total mentions	PCO concepts
Care experience	Healthcare professional communication	93	11.7%	Characteristics of communication: consistent; comprehensible; honest; kin; mindful; respectful; easy; positive; comprehensive what is communicated: hope; guidance; advice; answer to questions; options; bedside manner
	Care access	76	9.5%	Access to: support groups; treatment; mental health support; coordinated care, home care; specialist; doctors; oncologist, preventative tests; second opinion; multiple healthcare professionals; accessible care; timely care; nurses; alternative medicine; nutritionist; primary care; social workers
	Information access	61	7.7%	Knowledge of: how to manage health; about disease; what to ask; disease prognosis; who to contact; support services; answers to questions; cancer recurrence; understanding genetic basis of disease; information; disease cause; symptoms; rationale of tests Information overwhelm
	Healthcare team dynamics	9	1.1%	Healthcare team rapport; trust in healthcare provider; care team stability; communication between healthcare professionals; healthcare team approach to care; welcoming environment
	Perceived bias in care	5	0.6%	Impact of race on treatment of patient; impact of age on treatment of patient; impact of gender on treatment of patient
	Shared decision making	5	0.6%	Inclusion of caregiver in decision making; patient and healthcare provider working as a team; patient involvement in treatment planning; shared decision making
	Satisfaction & quality of care	2	0.3%	Quality of treatment; quality of treatment location
Psychosocial	Coping Abilities	50	6.3%	Maintaining: positivity; hope; stress; sense of normalcy; things that bring you joy; distractions; optimism; privacy; strength; acceptance of limitations. Cognitive avoidance Managing fear; managing mental health
	Impact on family/relationships	30	3.8%	Impact on family and loved ones, ability to be there for children/grandchildren, difficulty communicating about illness with loved ones, burden on family and loved ones, time with family and loved ones, emotional impact on family and loved ones, family well‐being, pressure from family and loved ones
	Anxiety	25	3.1%	Worry about: family and loved ones; disease; genetic risk; treatment; worst case scenarios; chemo effects; job; treatment efficacy Anxiety waiting for prognosis.
	Support network	24	3.0%	Support from: from family; from loved ones; from friends; from doctor, for caregiver; for loved ones; for children; having a support system.
	Distress	18	2.3%	Existential distress, fear of death, stress, fear, shock, stress about waiting, frustration, panic
	Involvement of close others	14	1.8%	A supportive person to attend appointments with; A supportive person helping with communication and understanding at appointments; caregiver and patient working as a team
	Self‐advocacy	11	1.4%	Self‐advocacy; advocating for access to care; prioritising self
	Emotional well‐being	9	1.1%	Emotional expression; emotional well‐being; emotional health; emotional support
	Social well‐being	6	0.8%	Ability to have a social life; social isolation
	Self‐image	5	0.6%	Forgetting about self; feeling comfortable in own body; sense of self
	Spiritual well‐being	5	0.6%	Leaning into spirituality; having faith; spiritual health; spiritual support
	Depression	4	0.5%	Depression
	Psychological well‐being	1	0.1%	Struggling with mental health
	Stigma	1	0.1%	Shame about diagnosis
Treatment	Treatment understanding	51	6.4%	Knowledge of: treatment side effects; treatment plan; treatment options; treatment impact; treatment risks; treatment alternatives; repercussions of not having treatment; treatment limitations; treatment outcomes; concise treatment information; how to prevent side effects; treatment efficacy; treatment type
	Treatment side effects	16	2.0%	Treatment side effects; side effect duration; physical treatment side effects; psychological treatment side effects
	Treatment timing	14	1.8%	Treatment: recovery time; duration; timing; frequency
	Treatment options	11	1.4%	Having multiple treatment options
	Treatment Impact	10	1.3%	Physical impact of treatment; impact of treatment; impact of treatment on daily life; impact of different treatments; long‐term impact of treatment; risk of treatment
	Treatment autonomy	8	1.0%	Ability to stop treatment; healthcare professionals respecting decision to stop treatment; deciding on treatment for self
	Treatment efficacy	8	1.0%	Treatment efficacy; positive surgery outcome; success of treatment; surgery being curative
	Treatment schedule	6	0.8%	Flexible treatment schedule; treatment sequencing; balanced treatment schedule; consistent treatment schedule
Lifestyle (life domains affected by cancer and its care)	Finances	40	5.0%	Insurance coverage, cost of treatment, financial planning, financial support, loss of income, cost of care, cost of transportation, financial worries, cost of childcare, financial impact on family, out of pocket costs
	Daily life	21	2.6%	Arranging childcare, lifestyle changes, time at home, managing schedule, putting aside personal life, help with chores, impact of diagnosis on daily life
	Quality of life	19	2.4%	Quality of life, living life fully, prioritising life experience
	Physical well‐being	6	0.8%	Physical health, physical well‐being, exercise, physical activity
	Life planning	5	0.6%	Ability to plan for different future scenarios, getting affairs in order, ability to make long‐term plans
	Weight/Nutrition	3	0.4%	Maintaining weight, weight loss, weight gain
	Diet	2	0.3%	Diet, dietary needs
	Appetite	1	0.1%	Loss of appetite
	Fertility	1	0.1%	Fertility
	Sleep	1	0.1%	Sleep
Healthcare utilisation	Wait times	22	2.8%	Wait times, wait time for diagnosis, wait time for treatment, wait time for healthcare provider, wait time for social worker, support during wait times
	Referrals	19	2.4%	Referral to support groups, referral to a dietician, referral to mental health support, referral to palliative care, referral to a nutritionist, referrals from oncologist
	Barriers to care	8	1.0%	Regional barriers to care, overcrowded hospital, Bureaucratic barriers to care, barriers to care for immigrants
	End of Life Care	8	1.0%	Advanced care planning, Medical assistance in dying (MAID), palliative care, palliative care planning
	Hospitalisation	6	0.8%	Length of hospital stay
Functional status (capacity to perform activities)	Work function	15	1.9%	Time off work, ability to work, loss of job
	Activities of daily living	10	1.3%	Impact on daily life, ability to drive, ability to engage in activities of daily living, ability to exercise
	Physical function	8	1.0%	Ability to travel, mobility, physical strength, need for physical help
	Personal independence	2	0.3%	Independence, sense of autonomy
Symptoms	Cosmetic	8	1.0%	Hair loss, physical appearance
	Fatigue	4	0.5%	Fatigue, low energy
	Pain	4	0.5%	Pain
	Dermatologic	2	0.3%	Translucent skin, rash
	Nausea/Vomiting	2	0.3%	Vomiting, nausea
	Bowel functions/Habits	1	0.1%	Diarrhoea
	Weakness	1	0.1%	Weakness

*Note:* Each row represents one PCO nested under its parent cluster. The right‐hand column lists all PCO concepts contributing to that PCO.

**Figure 3:**
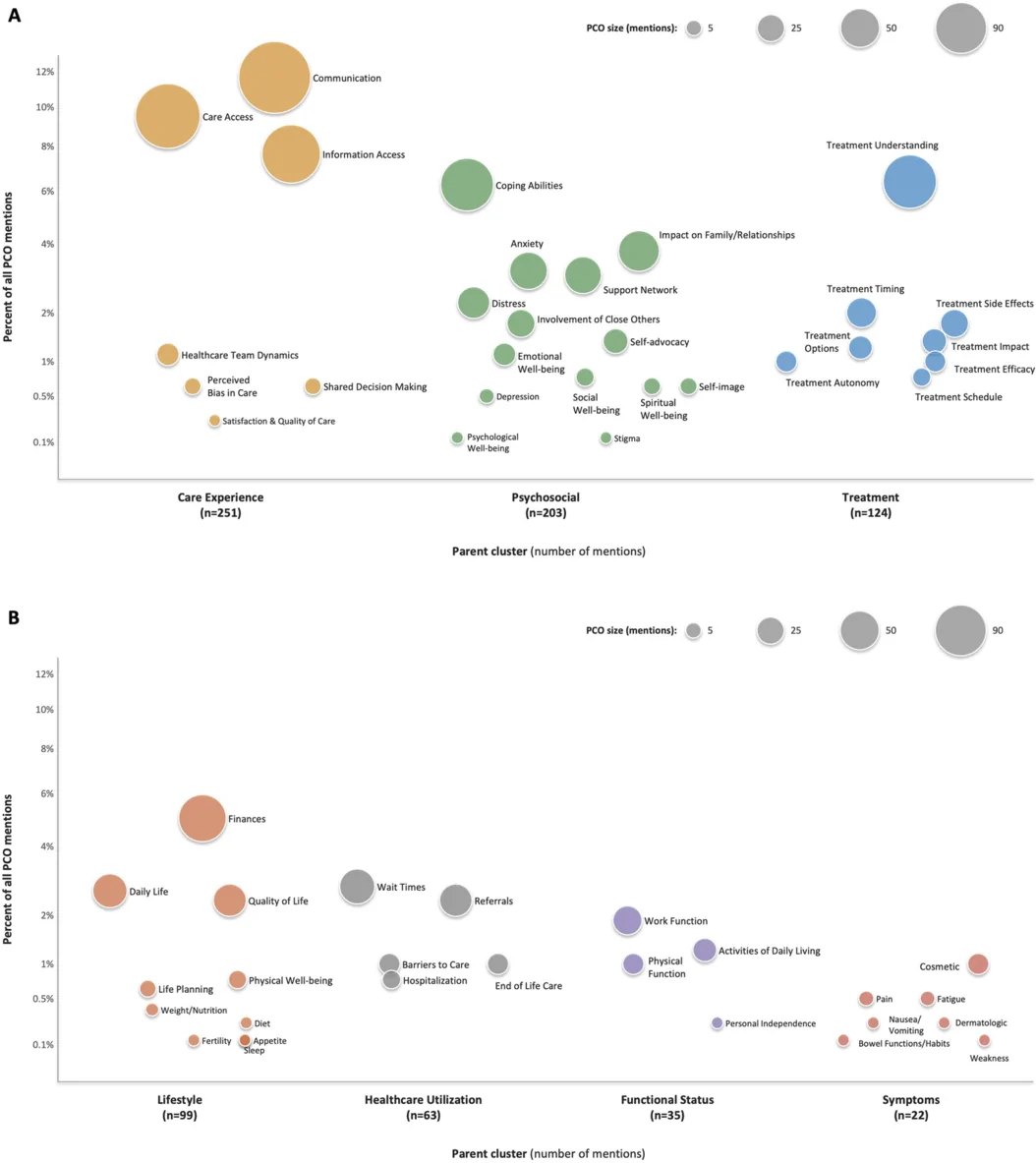
Distribution of patient‐centred outcomes (PCOs) and frequency of mentions, across parent clusters of care experience, psychosocial and treatment (A), and of lifestyle, healthcare utilisation, functional status and symptoms (B). Each bubble represents one PCO. Bubble area is proportional to the number of mentions.

The main PCO mentioned varied by type of participants (Figure 3). Patients most often brought up psychosocial PCOs (including coping abilities, self‐image, and spiritual well‐being). Care partners most often mentioned functional status and symptom PCOs (including activities of daily living, fatigue). Both patients and care partners uniformly raised treatment‐related PCOs (including treatment understanding, treatment side effects and treatment options).

The six most frequently mentioned PCOs are described below, organised by parent cluster, with illustrative participant quotes.

### 3.3 Care Experience Cluster

Seven PCOs were nested under care experience. Communication, care access, and information access together accounted for 28.9% of all mentions.

*Communication* (93 mentions) comprised 17 concepts describing provider–patient exchanges, most frequently consistency, comprehensibility, communication of expectations, and honesty. Participants described honesty and consistency across encounters as central to planning their lives:

I think the most important thing is the truth… The reality of the situation. I don't necessarily want all the gory details, but…I want the truth…so that I can plan my life out.

(P14)

If you don't ask, they don't tell… I feel like they're holding onto these resources and will only give certain things to you if you know that it's available.

(P5)

*Care access* (76 mentions) encompassed 17 concepts, most often access to support groups, access to treatment, access to mental health support, and coordinated care. Participants described both timeliness of treatment initiation and availability of psychosocial support as core components:

I would be worried about how fast I can get to the start of any of the treatments or procedures or things that are going to help… it felt to me like there was a real barrier to getting in the front door.

(P14)

I could've just used someone to talk to… maybe a volunteer, maybe a patient… this just happened to me, and I'm barely processing it, and I don't know how I'm supposed to exist throughout my day in my own brain right now.

(P23)

*Information access* (61 mentions) included 14 concepts, most often knowledge of how to manage one's health, knowledge about the disease itself, and knowing what to ask. Participants emphasised actionable information about lifestyle modification and recurrence risk:

They need to be educated on how to change their lifestyle… any factor that could've contributed to the cancer… whether it's environmental… getting into an exercise routine… basically doing what you can from all angles to prevent it, prevent the cancer from returning.

(P21)

A patient really deserves to know the accurate recurrence rates. But also any experimental or future drugs or things in progress that might come out that people should wait for or can sign up for… that's also really important.

(P19)

### 3.4 Treatment Cluster

Eight PCOs were nested under treatment. Treatment understanding was the most frequently mentioned within this cluster with 51 mentions. It comprised 13 concepts, most often knowledge of treatment side effects, knowledge of the treatment plan, and knowledge of treatment options. Participants described wanting explicit accounting of the downstream consequences of each option:

I don't think they were in the business of doing the nitty‐gritties. They were only telling you what the treatment was, not what it might do to you or what was involved.

(P2)

Probably the doctors don't want to tell you the side effects, or what are some of the nasty things that could happen, but the thing is, it would be better if they did that.

(CP17)

### 3.5 Psychosocial Cluster

Fourteen PCOs were nested under psychosocial, with coping abilities being mentioned most often (50 mentions). It included 13 concepts, including maintaining positivity, maintaining hope, and managing stress. Participants described adaptation to a changed life and the preservation of normalcy:

I'd like to add to the importance of staying positive… just to keep your head up and to maintain that positivity and just try and find something good out of something bad that's been handed to you.

(CP4)

Am I gonna be able to pick up and drop off my kid some of the days? Am I gonna be able to hang out and have dinner with my family? Am I gonna be able to maintain some normalcy?

(P23)

### 3.6 Lifestyle Cluster

Ten PCOs were nested under lifestyle; finances was the most frequently mentioned (40 mentions) and encompassed direct medical costs, out‐of‐pocket expenses, transportation, childcare, and loss of income. Participants described the financial implications of illness as a factor shaping treatment decisions themselves:

I don't have an income. I'm not 65, so I don't have my old‐age pension. And it, that terrified me. I thought, well, maybe I don't do treatment. But I have grandkids, you know…

(P8)

What are our finances? What can we do? We have mortgages, lots of things. So it affects the economics of the person, the relationships of that person, and their whole, whatever patterns they had in their previous, before they found out, those are all gone.

(CP1)

## 4 Discussion

From these focus groups, we compiled a list of PCOs meaningful to patients and care partners with lived experience of GI cancer. We identified 254 PCO concepts which we then consolidated into 55 PCOs distributed across 7 parent clusters: care experience, psychosocial, treatment, lifestyle, healthcare utilisation, functional status, and symptoms. Communication and care access were the PCOs most frequently brought up by participants, accounting for more than 20% of all PCO mentions.

Identifying which outcomes matter most to patients and care partners is critical, particularly given the differences between how patients and clinicians evaluate treatment success [26, 27, 28]. While clinicians often define success in terms of survival or disease response, patients and care partners may place greater emphasis on other outcomes [29]. Without understanding what those outcomes are, it is difficult to examine them in outcomes research, to communicate risk meaningfully, or to support shared decision‐making in clinical practice. Prior qualitative studies have catalogued PCOs within single cancer sites (such as gastric, upper GI or colorectal) or within a single supportive care domain such as communication or caregiver‐reported outcomes [30, 31, 32, 33, 34]. These efforts have focused on either patient or care‐partner perspectives in isolation, with only few studies bringing both into the analysis [30]. The GASTROS study developed a core outcome set for surgical gastric cancer trials and identified eight outcomes that are weighted toward clinical endpoints for trials (disease‐free survival, disease‐specific survival, recurrence, completeness of tumour removal, serious adverse events), with only overall quality of life and nutritional effects reflecting non‐clinical dimensions [35, 36]. Our focus groups were designed to assess the full range of outcomes that patients and care partners identify as meaningful across GI cancers. The resulting 55 PCOs included many that are not consistently captured in existing measures or core outcome sets for this population. By defining a broad and grounded set of PCOs not reflected in traditional measures, our work helped address the disconnect between what patients and care partners value and what is currently measured in GI cancer care.

Participants mentioned a range of PCOs that are not uniformly captured by instruments currently used in GI cancer research and care. Some of these PCOs map onto familiar constructs that have already been operationalized for use in practice and research. For instance, treatment side effects, have long been a core outcome in oncology research and validated symptom‐based instruments are well established [37]. Financial strain can be captured by validated instruments such as the COmprehensive Score for financial Toxicity (COST) measure [37, 38]. For these PCOs, our work confirms that patients and care partners consider them meaningful enough to bring them up unprompted, which reinforces their place in any clinical assessments, discussions, and research for GI cancer.

Other identified PCOs are only partially captured by existing instruments. Communication has been assessed in GI cancer care using tools such as the MISS‐21 and PSQ‐18, which address comprehensibility, kindness and respect, but not the consistency and honesty of communication across the care trajectory that participants emphasised [39, 40, 41, 42, 43, 44, 45]. Access to care overlaps partially with items in the CASUN and SCNS‐SF34 (e.g., access to support groups, psychological services, and specialists), but not fully [46, 47, 48, 49, 50, 51, 52]. Other PCOs raised frequently by participants, including self‐advocacy, perceived bias in care, maintaining positivity, and preserving a sense of normalcy, have little operationalization in the GI cancer literature. These will require further work to define how they may be operationalized and tested for integration into structured outcomes research or clinical practice.

Identifying PCOs meaningful to patients and care partners is a critical first step toward bridging the gaps about traditional clinical outcomes, but its value depends on how effectively these outcomes can be translated into individualised, clinically relevant information. Patients with GI cancers have reported that tailored estimates of outcomes with treatment are more relevant than generic, population‐based ones [53, 54]. Validated prediction models that synthesise complex clinical data into individualised estimates can support shared decision‐making and enhance care experience when aligned with patient values [55, 56, 57, 58, 59, 60]. The PCOs identified in this work provide a patient‐ and care‐partner‐informed starting point for which outcomes such tools should target. Routine assessment of all 55 PCOs is unlikely to be feasible within clinical workflows, nor was it the intent of this work; the list constitutes a comprehensive inventory rather than a measurement proposal. Future work will combine the data herein with results of a scoping review of PCOs reported in the published GI cancer literature to inform an international modified Delphi consensus process with patients, care partners, and healthcare professionals, aimed at prioritising PCOs most suitable for measurement and integration into future prediction and risk‐communication tools for GI cancer care, in the form of a core outcomes set [16, 36].

PCO mentions differed across patients and care partners. Patients most often raised PCOs within the psychosocial cluster, likely reflecting the emotional and identity‐related challenges of living with a GI cancer diagnosis, whereas care partners more frequently highlighted functional status, consistent with their role in managing the practical and physical demands of caregiving [61, 62, 63]. Treatment‐related PCOs were a mutual concern, indicating that the treatment journey remained a central point of impact for both groups. This divergence aligns with prior dyadic literature showing low concordance between patient and caregiver concerns [64, 65]. Outcomes derived from this work should therefore retain distinct patient‐facing and care‐partner‐facing components rather than collapsing the two perspectives.

This study has limitations. First, the sample was drawn from a single tertiary cancer centre in an urban setting, with most participants identifying as female and Caucasian. Patients and care partners in rural or remote settings report distinct healthcare experiences, access barriers, and outcome priorities compared with urban counterparts, and the PCOs identified here may therefore under‐represent considerations that are salient in those contexts [66, 67]. The female predominance in our sample reflects the demographic composition of care partners in oncology more broadly, but means that gendered patterns of caregiving and symptom reporting are likely over‐represented relative to male perspectives [68, 69]. The Caucasian predominance similarly limits the extent to which our findings reflect experiences shaped by racial and ethnic disparities in cancer care, which are well documented [70, 71]. The 55 PCOs identified here should be regarded as a starting point that requires confirmation and refinement in samples intentionally enriched for underrepresented groups, including rural, male, and non‐Caucasian patients and care partners. Second, our analytic approach weights PCOs by mention frequency. The patterns described here reflect *how often* each PCO surfaced, not necessarily how heavily it was weighted by individual participants. In addition, PCO salience likely varies along the cancer trajectory; our design compiled outcomes across the continuum but did not compare phases of care. The planned subsequent Delphi process is designed in part to address this limitation by asking participants to rate the relative importance of identified PCOs. The taxonomy presented here also reflects the interpretive decisions made during coding and consolidation; alternative groupings are possible, and other research teams approaching the same data might produce a different hierarchy. Third, some focus groups included as few as two participants. Smaller focus groups are an established variant in qualitative health research and can be beneficial for sensitive topics by allowing greater depth of individual contribution while retaining the interactive, participant‐driven discussion that distinguishes focus groups from interviews [72, 73]. However, the smallest groups may have functioned closer to dyadic interviews, with less of the group synergy of larger focus groups.

## 5 Conclusion

In focus groups with patients and care partners with lived experience of GI cancer, we identified 254 PCO concepts that we consolidated into 55 PCOs within seven parent clusters. These include concepts that are not consistently represented in existing instruments or core outcome sets for this population. Patients and care partners differed in which clusters they raised most frequently, with psychosocial concepts more often raised by patients and functional status more often raised by care partners, indicating that future tools should retain both perspectives rather than collapse them. As a next step, these PCOs will be prioritised through a Delphi process involving patients, care partners, and healthcare professionals to develop a core outcome set, which will in turn inform the development of risk‐prediction and communication tools aligned with what patients and care partners in GI cancer care value.

## Author Contributions

**Anna Gombay:** conceptualisation, investigation, data curation, project administration, methodology, writing – review and editing, writing – original draft, formal analysis. **Anna Ding:** conceptualisation, investigation, writing – review and editing, writing – original draft, project administration, methodology, formal analysis, data curation. **Alyson Mahar:** conceptualisation, investigation, methodology, writing – review and editing, funding acquisition, validation. **Amy T. Hsu:** conceptualisation, investigation, funding acquisition, methodology, validation, writing – review and editing. **Lesley Gotlib Conn:** conceptualisation, investigation, methodology, writing – review and editing, validation, formal analysis, supervision. **Jessica Armah:** resources, project administration, methodology, writing – review and editing. **Ekaterina Kosyachkova:** conceptualisation, investigation, validation, writing – review and editing, formal analysis. **Julie Deleemans:** conceptualisation, investigation, validation, writing – review and editing, formal analysis. **Claire Ludwig:** conceptualisation, investigation, validation, writing – review and editing, formal analysis. **Allia Karim:** conceptualisation, investigation, validation, writing – review and editing, formal analysis. **Natalie Coburn:** conceptualisation, investigation, methodology, funding acquisition, writing – review and editing, resources, validation. **Julie Hallet:** conceptualisation, investigation, funding acquisition, methodology, validation, writing – review and editing, resources, writing – original draft, visualisation.

## Ethics Statement

This study was approved by the Research Ethics Board of Sunnybrook Health Sciences Centre (#5195) and was conducted in accordance with the Declaration of Helsinki.

## Consent

All participants provided written informed consent prior to participation.

## Conflicts of Interest

JH has received speaking honoraria from Ipsen and Novartis and holds the Canada Research Chair in Patient‐Centred & Quality Cancer Surgery.

## Declaration

Claude, an AI assistant developed by Anthropic, was used to support copyediting and figure generation that was reviewed and verified by the authors; all study design, data collection, data analysis and interpretation, and reporting were conducted originally by the authors.

## Supporting information


Supporting File 1

Supporting File 2

## Data Availability

The data that support the findings of this study are available on request from the corresponding author. The data are not publicly available due to privacy or ethical restrictions. The data that support the findings of this study are available from the corresponding author upon reasonable request. Individual focus group transcripts are not publicly available to protect participant privacy.
