# Supplementary material for: Outcomes That Matter in Gastrointestinal Cancer Care: A Focus Group Study of Patient‐Centred Outcomes Identified by Patients and Care Partners
Source: Health Expect. 2026 Aug 21;29(4):e70835. doi: 10.1111/hex.70835 (PMC13495986; doi:10.1111/hex.70835)
Supplement: Supplementary file 1 [file HEX-29-e70835-s001.docx]

**Table S1. GRIPP2 short-form checklist for reporting patient and public involvement (online only)**

| **GRIPP2-SF item** | **Section reported** | **Summary of reporting** |
| --- | --- | --- |
| **1. Aim**  Report the aim of PPI in the study | Methods — Patient and public involvement | Three patient partners with lived experience of cancer were engaged as co-investigators from study inception, with the aim of grounding the study goals, design, conduct, and interpretation in lived experience. |
| **2. Methods**  Provide a clear description of the methods used for PPI in the study | Methods — Patient and public involvement | Patient partners contributed to the development and sequencing of the focus group guide, advised on the language of recruitment materials, and refined how PCOs were defined, identified, and categorized throughout analysis. Patient partners were members of the research team and did not participate in focus groups or transcript coding. |
| **3. Study results**  Report the results of PPI in the study, including both positive and negative outcomes | Methods — Patient and public involvement; Discussion | Partner input shaped the focus group guide to allow sufficient time for domains most relevant to patients, ensured recruitment terminology was clear, respectful, and sensitive, and broadened the conceptualization of what was counted as a PCO during extraction and clustering. |
| **4. Discussion and conclusions**  Comment on the extent to which PPI influenced the study overall; describe positive and negative effects | Methods — Patient and public involvement; Discussion (limitations) | Patient partner involvement influenced the study substantively, most notably the breadth of outcomes retained as PCOs. The limitations acknowledge that the resulting taxonomy reflects the interpretive decisions of the research team, of which patient partners were members. |
| **5. Reflections / critical perspective**  Comment critically on the study, reflecting on things that went well and those that did not | Data Supplement (this table) | Engagement from inception worked well for developing defining the research question and study design, developing the guide and recruitment materials, and for data interpretation. Patient partners were not involved in transcript coding which preserved independent extraction but limited their influence early in analytic decisions. |

PPI: patient and public involvement; PCO: patient-centred outcome.

Checklist items from Staniszewska S, Brett J, Simera I, et al. GRIPP2 reporting checklists: tools to improve reporting of patient and public involvement in research. BMJ. 2017;358:j3453.
