# Supplementary material for: Outcomes That Matter in Gastrointestinal Cancer Care: A Focus Group Study of Patient‐Centred Outcomes Identified by Patients and Care Partners
Source: Health Expect. 2026 Aug 21;29(4):e70835. doi: 10.1111/hex.70835 (PMC13495986; doi:10.1111/hex.70835)
Supplement: Supplementary file 2 [file HEX-29-e70835-s002.docx]

**Data Supplement**

**Outcomes that matter – patient-centred outcomes identification by patients and care partners focus groups in gastrointestinal cancer care**

**Fig S1. Hypothetical patient personas (Jane & John) (online only)**

*Photos of hypothetical individuals – AI generated.*
